# Oculo-facio-cardio-dental syndrome in a girl and her mother

**DOI:** 10.4103/0971-6866.73416

**Published:** 2010

**Authors:** Sudha Rudrappa, Rajendra Kumar, G. S. Kumar

**Affiliations:** Department of Pediatrics, Mysore Medical College and Research Institute, Mysore, Karnataka, India

**Keywords:** Congenital heart defects, dental anomalies, facial dysmorphism, X-linked dominant inheritance

## Abstract

Congenital heart defects are known to be associated with facial dysmorphism and other congenital anomalies. Oculo-facio-cardio-dental (OFCD) syndrome is one such rare multiple congenital anomaly syndrome inherited as an X-linked dominant condition characterized by congenital cataracts, multiple minor facial dysmorphic features, congenital heart defects and dental anomalies. It is unrecognized by many medical and dental professionals. Only 21 cases have been reported so far. This syndrome is often misrecognized as rubella embryopathy because of association of congenital cataract with cardiac anomalies. It is usually the orthodontists who diagnose the syndrome based on typical findings on dental panoramic radiographs. But we suspected our patient to be having OFCD syndrome based on typical facial dysmorphism, ocular and cardiac defects, and finally it was confirmed after noticing typical dental radiographic findings.

## Introduction

Presentation of cardiac defects in association with other developmental defects as a part of genetic syndrome is well known. We report a rare genetic syndrome in which cardiac defect is associated with characteristic ocular, facial, and dental anomalies. Such an association has been named as oculo-facio-cardio-dental (OFCD) syndrome by Obwegeser and Gorlin,[1] and its genetic basis has been discovered recently. Without observing dental anomalies, association of cardiac defect and congenital cataracts is often misrecognized as a result of rubella embryopathy.[2] This makes the diagnosis of OFCD difficult for medical specialists, and the syndrome often remains unrecognized. Thus, only 21 cases have been reported worldwide so far.

## Case Report

A 6-year-old girl, born of a second degree consanguineous marriage, presented with history of non-productive cough of 15 days duration, orthopnea and prominent neck pulsations. There was no history of fever. Her birth history was uneventful and she had an elder sister who was said to be healthy. There was mild motor and language developmental delay.

**Figure 1:**
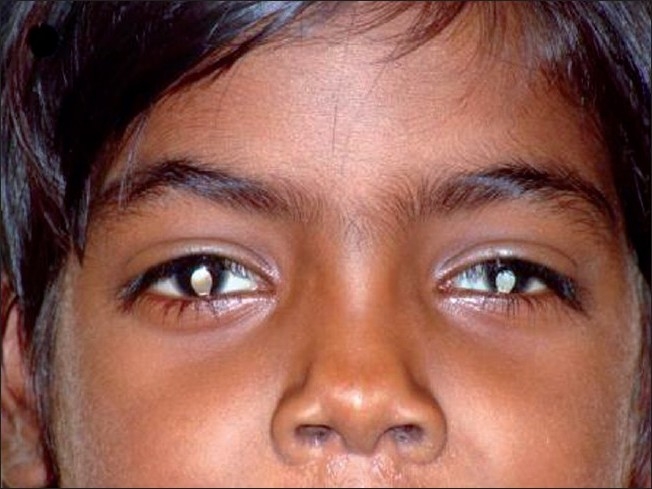
Ocular and facial features of index case.

On examination, she had retarded growth (weight and height being less than 3^rd^ centile). Both tachycardia and tachypnea were present. Her face was dysmorphic, long and narrow, with high nasal bridge, broad nasal tip with separated nasal cartilages, laterally curved and thick eyebrows, long philtrum and simple left ear [Figure 1]. A mild exotropia, bilateral typical complete coloboma, left-sided microcornea with cortical cataract and searching nystagmus were present in both the eyes. Her vision could not be assessed due to nystagmus. She had all 20 temporary teeth erupted, but permanent tooth had not yet erupted. She also had a small reducible umbilical hernia and fifth finger clinodactyly of both hands (left side more obvious than right side). Cardiac examination revealed a wide and fixed spilt S_2_, loud P_2_, S_3_ gallop at apex and a grade III/VI ejection systolic murmur over pulmonary area. Fine crepitations and rhonchi were heard on lung bases of both the sides.

Her investigations were as follows:

Hb: 11.6 g/dl; TC: 11,300 cells/mm^3^; DC: N_54_L_33_M_13_, peripheral smear showed normocytic, normochromic anemia; erythrocyte sedimentation rate (ESR): 40 mmHg; urine examination was unremarkable. Blood culture had no growth. A mild cardiomegaly was present on chest X-ray, with pulmonary plethora and patchy pneumonitis in left middle zone. 2D-echocardiography revealed a large ostium secondum atrial septal defect (ASD), (17 mm in size) with dilated right atrium and right ventricle. The karyotype was 46, XX.

**Figure 2:**
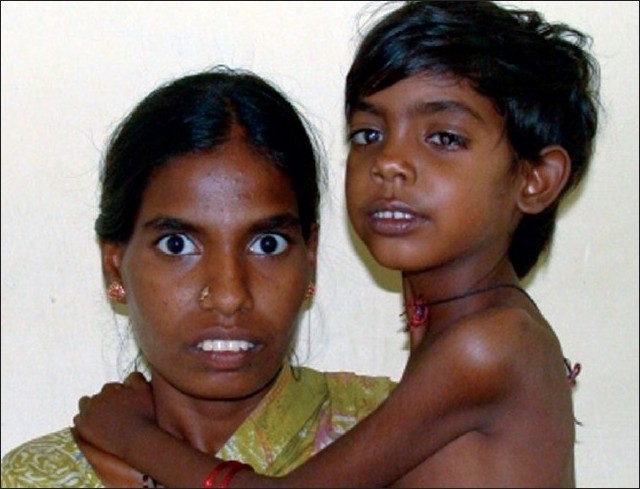
Facial features of mother and the child.

Her mother was also noticed to have bilateral complete typical coloboma and fifth finger clinodactyly of both hands (left side more obvious than right side). She also had long narrow face, high nasal bridge, thick eyebrows and dental malocclusion [Figure 2]. Cardiac examination was unremarkable.

Based on typical facial features and ocular findings associated with ASD, we made the diagnosis of OFCD. Further, a dental panoramic radiograph of mother revealed canine teeth radiculomegaly.

The child was treated with IV antibiotics, IV frusemide and digoxin. The child responded well to treatment. She was discharged with advice of regular follow-up, early closure of ASD, regular ophthalmic and dental evaluation.

## Discussion

OFCD syndrome is a very rare multiple congenital anomaly which has an estimated incidence of less than one in 1 million people. Obwegeser and Gorlin (1997), who referred to this condition as the OFCD syndrome, stated that Hayward (1980) was probably the first to report the association of congenital cataracts and radiculomegaly of the canine teeth.[1] The syndrome is usually diagnosed retrospectively, often by orthodontists, when they notice typical dental anomalies (persistence of primary teeth, radiculomegaly, etc) and there will be history of surgery for ASD and cataract with facial dysmorphism.[2,3] Table 1 highlights the features of OFCD syndrome so far observed.

**Table 1:** List of various abnormalities described in Occulo-facio-cardio-dental syndrome

Ocular findings	Facial findings	Cardiac findings	Dental findings	Skeletal findings	Other findings
Congenital cataract*	Septate nasal cartilage*	Unresolved heart murmur	Delayed/persistent/unerupted dentition	Hammer toes	Mental retardation*
Microphthalmia/microcornea*	High nasal bridge*†	Septal defects (ASD*, VSD)	Root radiculomegaly (secondary teeth)	Second-third toe syndactyly	Cerebral atrophy ADHD
Coloboma *† Ptosis	Long narrow face*†	Patent ductus arteriosus	Hypodontia/duplication/fusion (secondary teeth)	Radioulnar synostosis	Hearing impairment
Secondary glaucoma	Palate/uvula anomalies	Valve incompetency	Enamel defects	Limited supination at wrist	Poor feeding
Lens dislocation	Simple ears*	Pentalogy of Fallot	Root dilacerations	Lordosis/scoliosis	Vomiting/reflux
Optic disk dysplasia		Dextrocardia	Malposistion and malocclusion†	Vertebral fusion	Asplenia
Phthisis bulbi		Double outlet right ventricle		Short fingers *†	Vesicoureteral reflux
Iris synechia					
Retinal detachment					
Laterally curved and thick eyebrows*†					

* Findings which were present in our index case

† Findings which were present in mother

This condition is inherited in an X-linked dominant pattern.[4] Mutation in *BCOR* gene located on Xp11.4 is responsible for the syndrome.[5,6] However, exact function of this gene is unknown. In females (who have two X chromosomes), a mutation in one of the two copies of the gene in each cell is sufficient to cause the disorder. Some cells produce a normal amount of BCOR co-repressor protein and other cells produce none. The resulting overall reduction in the amount of this protein leads to the signs and symptoms of OFCD syndrome. In males (who have only one X chromosome), mutations result in a total loss of the BCOR co-repressor protein. Lack of this protein appears to be lethal very early in development, and hence no males are born with OFCD syndrome.

## Conclusion

To the best of the authors’ knowledge, this is fourth report of a mother–daughter vertical transmission.[7] It is the typical facial and ocular features in the child and the mother that led us to make the diagnosis of OFCD syndrome. Early diagnosis of such syndromes allows better patient management and gives scope for genetic counseling.
